# Supplementary material for: Homology-Based Image Processing for Automatic Classification of Histopathological Images of Lung Tissue
Source: Cancers (Basel). 2021 Mar 10;13(6):1192. doi: 10.3390/cancers13061192 (PMC8001245; doi:10.3390/cancers13061192)
Supplement: Supplementary file 1 [file cancers-13-01192-s001.pdf]

*Supplementary Materials*

# Homology-Based Image Processing for Automatic Classification of Histopathological Images of Lung Tissue

**Mizuho Nishio, Mari Nishio, Naoe Jimbo and Kazuaki Nakane**

1. A

$b_0 = 2$, $b_1 = 1$

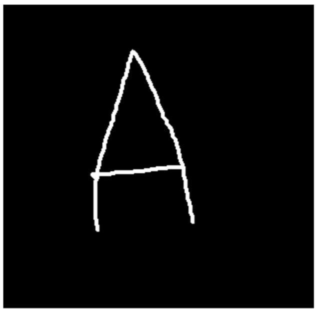

2. a

$b_0 = 2$, $b_1 = 1$

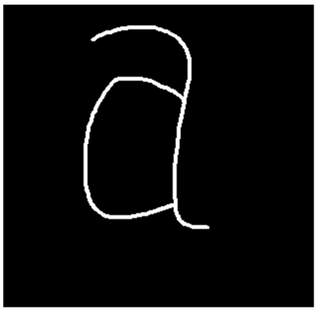

3. B

$b_0 = 3$, $b_1 = 1$

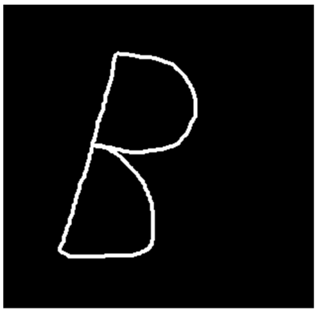

4. b

$b_0 = 2, b_1 = 1$

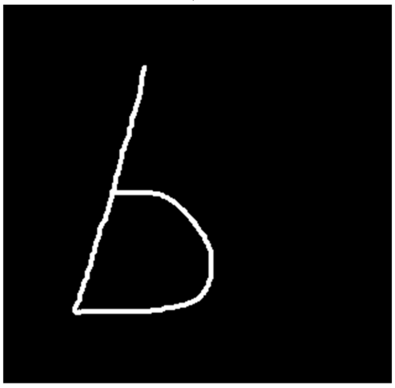

5. I

$b_0 = 1, b_1 = 1$

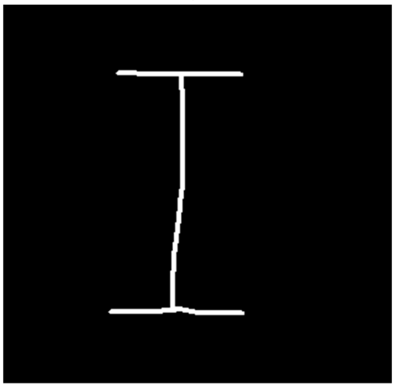

6. i

$b_0 = 1, b_1 = 2$

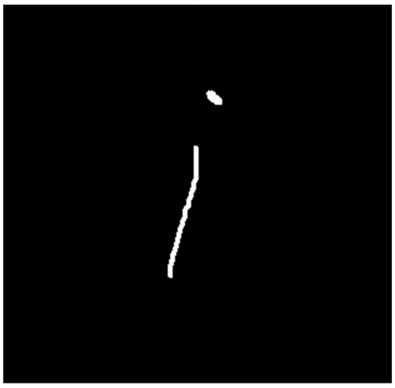

**Figure S1.** Binarized image of handwritten character and its Betti numbers. Note: Betti numbers of binarized image of handwritten character were calculated. In the images, $b_0$ corresponds to the number of black regions; $b_1$ corresponds to the number of white regions.

**Table S1.** Feature names of texture analysis.

| Number | Name of Feature |
| --- | --- |
| 1 | original_firstorder_10Percentile |
| 2 | original_firstorder_90Percentile |
| 3 | original_firstorder_Energy |
| 4 | original_firstorder_Entropy |
| 5 | original_firstorder_InterquartileRange |
| 6 | original_firstorder_Kurtosis |
| 7 | original_firstorder_Maximum |
| 8 | original_firstorder_MeanAbsoluteDeviation |
| 9 | original_firstorder_Mean |
| 10 | original_firstorder_Median |
| 11 | original_firstorder_Minimum |
| 12 | original_firstorder_Range |
| 13 | original_firstorder_RobustMeanAbsoluteDeviation |
| 14 | original_firstorder_RootMeanSquared |
| 15 | original_firstorder_Skewness |
| 16 | original_firstorder_TotalEnergy |
| 17 | original_firstorder_Uniformity |
| 18 | original_firstorder_Variance |
| 19 | original_glcm_Autocorrelation |
| 20 | original_glcm_ClusterProminence |
| 21 | original_glcm_ClusterShade |
| 22 | original_glcm_ClusterTendency |
| 23 | original_glcm_Contrast |
| 24 | original_glcm_Correlation |
| 25 | original_glcm_DifferenceAverage |
| 26 | original_glcm_DifferenceEntropy |
| 27 | original_glcm_DifferenceVariance |
| 28 | original_glcm_Id |
| 29 | original_glcm_Idm |
| 30 | original_glcm_Idmn |
| 31 | original_glcm_Idn |
| 32 | original_glcm_Imc1 |
| 33 | original_glcm_Imc2 |
| 34 | original_glcm_InverseVariance |
| 35 | original_glcm_JointEnergy |
| 36 | original_glcm_JointEntropy |
| 37 | original_glcm_MCC |
| 38 | original_glcm_MaximumProbability |
| 39 | original_glcm_SumAverage |
| 40 | original_glcm_SumEntropy |
| 41 | original_glcm_SumSquares |
| 42 | original_glrlm_ShortRunEmphasis |
| 43 | original_glrlm_LongRunEmphasis |
| 44 | original_glrlm_GrayLevelNonUniformity |
| 45 | original_glrlm_GrayLevelNonUniformityNormalized |
| 46 | original_glrlm_RunLengthNonUniformity |
| 47 | original_glrlm_RunLengthNonUniformityNormalized |
| 48 | original_glrlm_RunPercentage |
| 49 | original_glrlm_GrayLevelVariance |
| 50 | original_glrlm_RunVariance |
| 51 | original_glrlm_RunEntropy |
| 52 | original_glrlm_LowGrayLevelRunEmphasis |
| 53 | original_glrlm_HighGrayLevelRunEmphasis |
| 54 | original_glrlm_ShortRunLowGrayLevelEmphasis |
| 55 | original_glrlm_ShortRunHighGrayLevelEmphasis |
| 56 | original_glrlm_LongRunLowGrayLevelEmphasis |

| | |
|---|---|
| 57 | original_glrlm_LongRunHighGrayLevelEmphasis |
| 58 | original_glszm_SmallAreaEmphasis |
| 59 | original_glszm_LargeAreaEmphasis |
| 60 | original_glszm_GrayLevelNonUniformity |
| 61 | original_glszm_GrayLevelNonUniformityNormalized |
| 62 | original_glszm_SizeZoneNonUniformity |
| 63 | original_glszm_SizeZoneNonUniformityNormalized |
| 64 | original_glszm_ZonePercentage |
| 65 | original_glszm_GrayLevelVariance |
| 66 | original_glszm_ZoneVariance |
| 67 | original_glszm_ZoneEntropy |
| 68 | original_glszm_LowGrayLevelZoneEmphasis |
| 69 | original_glszm_HighGrayLevelZoneEmphasis |
| 70 | original_glszm_SmallAreaLowGrayLevelEmphasis |
| 71 | original_glszm_SmallAreaHighGrayLevelEmphasis |
| 72 | original_glszm_LargeAreaLowGrayLevelEmphasis |
| 73 | original_glszm_LargeAreaHighGrayLevelEmphasis |
| 74 | original_gldm_SmallDependenceEmphasis |
| 75 | original_gldm_LargeDependenceEmphasis |
| 76 | original_gldm_GrayLevelNonUniformity |
| 77 | original_gldm_DependenceNonUniformity |
| 78 | original_gldm_DependenceNonUniformityNormalized |
| 79 | original_gldm_GrayLevelVariance |
| 80 | original_gldm_DependenceVariance |
| 81 | original_gldm_DependenceEntropy |
| 82 | original_gldm_LowGrayLevelEmphasis |
| 83 | original_gldm_HighGrayLevelEmphasis |
| 84 | original_gldm_SmallDependenceLowGrayLevelEmphasis |
| 85 | original_gldm_SmallDependenceHighGrayLevelEmphasis |
| 86 | original_gldm_LargeDependenceLowGrayLevelEmphasis |
| 87 | original_gldm_LargeDependenceHighGrayLevelEmphasis |
| 88 | original_ngtdm_Coarseness |
| 89 | original_ngtdm_Contrast |
| 90 | original_ngtdm_Busyness |
| 91 | original_ngtdm_Complexity |
| 92 | original_ngtdm_Strength |

**Table S2.** Validation accuracies of CAD with homology-based image processing in the private dataset.

| Normalization | Feature Selection | Image Resolutions | | | | Five-Category Accuracy | Optimal Machine Learning Algorithm |
|---|---|---|---|---|---|---|---|
| 0 | 0 | 128 × 128 | | | | 0.85 | 1 |
| 0 | 0 | 256 × 256 | | | | 0.795 | 6 |
| 0 | 0 | 256 × 256 | 128 × 128 | | | 0.835 | 6 |
| 0 | 0 | 512 × 512 | | | | 0.84 | 7 |
| 0 | 0 | 512 × 512 | 128 × 128 | | | 0.84 | 7 |
| 0 | 0 | 512 × 512 | 256 × 256 | | | 0.84 | 7 |
| 0 | 0 | 512 × 512 | 256 × 256 | 128 × 128 | | 0.86 | 7 |
| 0 | 0 | 1024 × 1024 | | | | 0.645 | 5 |
| 0 | 0 | 1024 × 1024 | 128 × 128 | | | 0.695 | 6 |
| 0 | 0 | 1024 × 1024 | 256 × 256 | | | 0.735 | 6 |
| 0 | 0 | 1024 × 1024 | 256 × 256 | 128 × 128 | | 0.765 | 7 |
| 0 | 0 | 1024 × 1024 | 512 × 512 | | | 0.73 | 6 |
| 0 | 0 | 1024 × 1024 | 512 × 512 | 128 × 128 | | 0.755 | 7 |
| 0 | 0 | 1024 × 1024 | 512 × 512 | 256 × 256 | | 0.76 | 6 |
| 0 | 0 | 1024 × 1024 | 512 × 512 | 256 × 256 | 128 × 128 | 0.77 | 7 |
| 0 | 1 | 128 × 128 | | | | 0.81 | 1 |
| 0 | 1 | 256 × 256 | | | | 0.9 | 6 |
| 0 | 1 | 256 × 256 | 128 × 128 | | | 0.845 | 6 |
| 0 | 1 | 512 × 512 | | | | 0.8 | 7 |
| 0 | 1 | 512 × 512 | 128 × 128 | | | 0.865 | 7 |
| 0 | 1 | 512 × 512 | 256 × 256 | | | 0.84 | 6 |
| 0 | 1 | 512 × 512 | 256 × 256 | 128 × 128 | | 0.835 | 7 |
| 0 | 1 | 1024 × 1024 | | | | 0.685 | 6 |
| 0 | 1 | 1024 × 1024 | 128 × 128 | | | 0.795 | 6 |
| 0 | 1 | 1024 × 1024 | 256 × 256 | | | 0.795 | 6 |
| 0 | 1 | 1024 × 1024 | 256 × 256 | 128 × 128 | | 0.81 | 6 |
| 0 | 1 | 1024 × 1024 | 512 × 512 | | | 0.785 | 6 |
| 0 | 1 | 1024 × 1024 | 512 × 512 | 128 × 128 | | 0.805 | 6 |
| 0 | 1 | 1024 × 1024 | 512 × 512 | 256 × 256 | | 0.805 | 6 |
| 0 | 1 | 1024 × 1024 | 512 × 512 | 256 × 256 | 128 × 128 | 0.815 | 6 |
| 1 | 0 | 128 × 128 | | | | 0.77 | 6 |
| 1 | 0 | 256 × 256 | | | | 0.795 | 6 |
| 1 | 0 | 256 × 256 | 128 × 128 | | | 0.84 | 6 |
| 1 | 0 | 512 × 512 | | | | 0.84 | 7 |
| 1 | 0 | 512 × 512 | 128 × 128 | | | 0.84 | 7 |
| 1 | 0 | 512 × 512 | 256 × 256 | | | 0.84 | 7 |
| 1 | 0 | 512 × 512 | 256 × 256 | 128 × 128 | | 0.86 | 7 |
| 1 | 0 | 1024 × 1024 | | | | 0.66 | 1 |
| 1 | 0 | 1024 × 1024 | 128 × 128 | | | 0.705 | 1 |
| 1 | 0 | 1024 × 1024 | 256 × 256 | | | 0.76 | 3 |
| 1 | 0 | 1024 × 1024 | 256 × 256 | 128 × 128 | | 0.765 | 7 |
| 1 | 0 | 1024 × 1024 | 512 × 512 | | | 0.73 | 6 |
| 1 | 0 | 1024 × 1024 | 512 × 512 | 128 × 128 | | 0.755 | 7 |
| 1 | 0 | 1024 × 1024 | 512 × 512 | 256 × 256 | | 0.76 | 0 |
| 1 | 0 | 1024 × 1024 | 512 × 512 | 256 × 256 | 128 × 128 | 0.775 | 0 |
| 1 | 1 | 128 × 128 | | | | 0.82 | 6 |
| 1 | 1 | 256 × 256 | | | | 0.865 | 6 |
| 1 | 1 | 256 × 256 | 128 × 128 | | | 0.88 | 6 |
| 1 | 1 | 512 × 512 | | | | 0.825 | 7 |
| 1 | 1 | 512 × 512 | 128 × 128 | | | 0.88 | 7 |
| 1 | 1 | 512 × 512 | 256 × 256 | | | 0.885 | 5 |
| 1 | 1 | 512 × 512 | 256 × 256 | 128 × 128 | | 0.835 | 7 |
| 1 | 1 | 1024 × 1024 | | | | 0.665 | 6 |
| 1 | 1 | 1024 × 1024 | 128 × 128 | | | 0.855 | 6 |
| 1 | 1 | 1024 × 1024 | 256 × 256 | | | 0.83 | 6 |
| 1 | 1 | 1024 × 1024 | 256 × 256 | 128 × 128 | | 0.8 | 6 |
| 1 | 1 | 1024 × 1024 | 512 × 512 | | | 0.765 | 6 |
| 1 | 1 | 1024 × 1024 | 512 × 512 | 128 × 128 | | 0.85 | 6 |
| 1 | 1 | 1024 × 1024 | 512 × 512 | 256 × 256 | | 0.86 | 6 |
| 1 | 1 | 1024 × 1024 | 512 × 512 | 256 × 256 | 128 × 128 | 0.86 | 6 |

**Table S3.** Validation accuracies of CAD with texture analysis in the private dataset.

| Normalization | Feature Selection | Image Resolutions | | | | Five-Category Accuracy | Optimal Machine Learning Algorithm |
|---|---|---|---|---|---|---|---|
| 0 | 0 | 128 × 128 | | | | 0.77 | 5 |
| 0 | 0 | 256 × 256 | | | | 0.715 | 6 |
| 0 | 0 | 256 × 256 | 128 × 128 | | | 0.72 | 7 |
| 0 | 0 | 512 × 512 | | | | 0.705 | 5 |
| 0 | 0 | 512 × 512 | 128 × 128 | | | 0.695 | 6 |
| 0 | 0 | 512 × 512 | 256 × 256 | | | 0.715 | 5 |
| 0 | 0 | 512 × 512 | 256 × 256 | 128 × 128 | | 0.71 | 5 |
| 0 | 0 | 1024 × 1024 | | | | 0.72 | 6 |
| 0 | 0 | 1024 × 1024 | 128 × 128 | | | 0.815 | 5 |
| 0 | 0 | 1024 × 1024 | 256 × 256 | | | 0.655 | 6 |
| 0 | 0 | 1024 × 1024 | 256 × 256 | 128 × 128 | | 0.74 | 5 |
| 0 | 0 | 1024 × 1024 | 512 × 512 | | | 0.65 | 5 |
| 0 | 0 | 1024 × 1024 | 512 × 512 | 128 × 128 | | 0.725 | 6 |
| 0 | 0 | 1024 × 1024 | 512 × 512 | 256 × 256 | | 0.69 | 6 |
| 0 | 0 | 1024 × 1024 | 512 × 512 | 256 × 256 | 128 × 128 | 0.665 | 5 |
| 0 | 1 | 128 × 128 | | | | 0.73 | 5 |
| 0 | 1 | 256 × 256 | | | | 0.81 | 2 |
| 0 | 1 | 256 × 256 | 128 × 128 | | | 0.75 | 6 |
| 0 | 1 | 512 × 512 | | | | 0.835 | 2 |
| 0 | 1 | 512 × 512 | 128 × 128 | | | 0.725 | 6 |
| 0 | 1 | 512 × 512 | 256 × 256 | | | 0.85 | 2 |
| 0 | 1 | 512 × 512 | 256 × 256 | 128 × 128 | | 0.725 | 6 |
| 0 | 1 | 1024 × 1024 | | | | 0.865 | 1 |
| 0 | 1 | 1024 × 1024 | 128 × 128 | | | 0.755 | 6 |
| 0 | 1 | 1024 × 1024 | 256 × 256 | | | 0.745 | 6 |
| 0 | 1 | 1024 × 1024 | 256 × 256 | 128 × 128 | | 0.735 | 6 |
| 0 | 1 | 1024 × 1024 | 512 × 512 | | | 0.71 | 6 |
| 0 | 1 | 1024 × 1024 | 512 × 512 | 128 × 128 | | 0.7 | 6 |
| 0 | 1 | 1024 × 1024 | 512 × 512 | 256 × 256 | | 0.73 | 5 |
| 0 | 1 | 1024 × 1024 | 512 × 512 | 256 × 256 | 128 × 128 | 0.74 | 6 |
| 1 | 0 | 128 × 128 | | | | 0.83 | 3 |
| 1 | 0 | 256 × 256 | | | | 0.77 | 3 |
| 1 | 0 | 256 × 256 | 128 × 128 | | | 0.78 | 4 |
| 1 | 0 | 512 × 512 | | | | 0.8 | 0 |
| 1 | 0 | 512 × 512 | 128 × 128 | | | 0.815 | 4 |
| 1 | 0 | 512 × 512 | 256 × 256 | | | 0.77 | 3 |
| 1 | 0 | 512 × 512 | 256 × 256 | 128 × 128 | | 0.8 | 4 |
| 1 | 0 | 1024 × 1024 | | | | 0.77 | 4 |
| 1 | 0 | 1024 × 1024 | 128 × 128 | | | 0.815 | 5 |
| 1 | 0 | 1024 × 1024 | 256 × 256 | | | 0.815 | 2 |
| 1 | 0 | 1024 × 1024 | 256 × 256 | 128 × 128 | | 0.81 | 4 |
| 1 | 0 | 1024 × 1024 | 512 × 512 | | | 0.805 | 1 |
| 1 | 0 | 1024 × 1024 | 512 × 512 | 128 × 128 | | 0.82 | 3 |
| 1 | 0 | 1024 × 1024 | 512 × 512 | 256 × 256 | | 0.835 | 3 |
| 1 | 0 | 1024 × 1024 | 512 × 512 | 256 × 256 | 128 × 128 | 0.825 | 4 |
| 1 | 1 | 128 × 128 | | | | 0.825 | 0 |
| 1 | 1 | 256 × 256 | | | | 0.81 | 6 |
| 1 | 1 | 256 × 256 | 128 × 128 | | | 0.83 | 4 |
| 1 | 1 | 512 × 512 | | | | 0.8 | 4 |
| 1 | 1 | 512 × 512 | 128 × 128 | | | 0.8 | 0 |
| 1 | 1 | 512 × 512 | 256 × 256 | | | 0.795 | 4 |
| 1 | 1 | 512 × 512 | 256 × 256 | 128 × 128 | | 0.8 | 4 |
| 1 | 1 | 1024 × 1024 | | | | 0.72 | 2 |
| 1 | 1 | 1024 × 1024 | 128 × 128 | | | 0.82 | 4 |
| 1 | 1 | 1024 × 1024 | 256 × 256 | | | 0.82 | 4 |
| 1 | 1 | 1024 × 1024 | 256 × 256 | 128 × 128 | | 0.82 | 4 |
| 1 | 1 | 1024 × 1024 | 512 × 512 | | | 0.79 | 4 |
| 1 | 1 | 1024 × 1024 | 512 × 512 | 128 × 128 | | 0.8 | 4 |
| 1 | 1 | 1024 × 1024 | 512 × 512 | 256 × 256 | | 0.82 | 3 |
| 1 | 1 | 1024 × 1024 | 512 × 512 | 256 × 256 | 128 × 128 | 0.815 | 4 |

**Table S4.** Validation accuracies of CAD with homology-based image processing in the public dataset.

| Normalization | Feature Selection | Image Resolutions | | | | Three-Category Accuracy | Optimal Machine Learning Algorithm |
|---|---|---|---|---|---|---|---|
| 0 | 0 | 96 × 96 | | | | 0.9810 | 7 |
| 0 | 0 | 192 × 192 | | | | 0.9907 | 7 |
| 0 | 0 | 192 × 192 | 96 × 96 | | | 0.9913 | 7 |
| 0 | 0 | 384 × 384 | | | | 0.9923 | 7 |
| 0 | 0 | 384 × 384 | 96 × 96 | | | 0.9923 | 7 |
| 0 | 0 | 384 × 384 | 192 × 192 | | | 0.9917 | 7 |
| 0 | 0 | 384 × 384 | 192 × 192 | 96 × 96 | | 0.9927 | 7 |
| 0 | 0 | 768 × 768 | | | | 0.9897 | 7 |
| 0 | 0 | 768 × 768 | 96 × 96 | | | 0.9910 | 7 |
| 0 | 0 | 768 × 768 | 192 × 192 | | | 0.9900 | 7 |
| 0 | 0 | 768 × 768 | 192 × 192 | 96 × 96 | | 0.9913 | 7 |
| 0 | 0 | 768 × 768 | 384 × 384 | | | 0.9927 | 7 |
| 0 | 0 | 768 × 768 | 384 × 384 | 96 × 96 | | 0.9907 | 7 |
| 0 | 0 | 768 × 768 | 384 × 384 | 192 × 192 | | 0.9900 | 7 |
| 0 | 0 | 768 × 768 | 384 × 384 | 192 × 192 | 96 × 96 | 0.9927 | 7 |
| 0 | 1 | 96 × 96 | | | | 0.9250 | 7 |
| 0 | 1 | 192 × 192 | | | | 0.9633 | 7 |
| 0 | 1 | 192 × 192 | 96 × 96 | | | 0.9740 | 7 |
| 0 | 1 | 384 × 384 | | | | 0.9710 | 7 |
| 0 | 1 | 384 × 384 | 96 × 96 | | | 0.9827 | 7 |
| 0 | 1 | 384 × 384 | 192 × 192 | | | 0.9740 | 7 |
| 0 | 1 | 384 × 384 | 192 × 192 | 96 × 96 | | 0.9807 | 7 |
| 0 | 1 | 768 × 768 | | | | 0.9687 | 7 |
| 0 | 1 | 768 × 768 | 96 × 96 | | | 0.9793 | 7 |
| 0 | 1 | 768 × 768 | 192 × 192 | | | 0.9783 | 7 |
| 0 | 1 | 768 × 768 | 192 × 192 | 96 × 96 | | 0.9810 | 7 |
| 0 | 1 | 768 × 768 | 384 × 384 | | | 0.9773 | 7 |
| 0 | 1 | 768 × 768 | 384 × 384 | 96 × 96 | | 0.9833 | 7 |
| 0 | 1 | 768 × 768 | 384 × 384 | 192 × 192 | | 0.9820 | 7 |
| 0 | 1 | 768 × 768 | 384 × 384 | 192 × 192 | 96 × 96 | 0.9847 | 7 |
| 1 | 0 | 96 × 96 | | | | 0.9810 | 7 |
| 1 | 0 | 192 × 192 | | | | 0.9907 | 7 |
| 1 | 0 | 192 × 192 | 96 × 96 | | | 0.9913 | 7 |
| 1 | 0 | 384 × 384 | | | | 0.9923 | 7 |
| 1 | 0 | 384 × 384 | 96 × 96 | | | 0.9923 | 7 |
| 1 | 0 | 384 × 384 | 192 × 192 | | | 0.9917 | 7 |
| 1 | 0 | 384 × 384 | 192 × 192 | 96 × 96 | | 0.9927 | 7 |
| 1 | 0 | 768 × 768 | | | | 0.9897 | 7 |
| 1 | 0 | 768 × 768 | 96 × 96 | | | 0.9910 | 7 |
| 1 | 0 | 768 × 768 | 192 × 192 | | | 0.9903 | 2 |
| 1 | 0 | 768 × 768 | 192 × 192 | 96 × 96 | | 0.9913 | 7 |
| 1 | 0 | 768 × 768 | 384 × 384 | | | 0.9927 | 7 |
| 1 | 0 | 768 × 768 | 384 × 384 | 96 × 96 | | 0.9907 | 7 |
| 1 | 0 | 768 × 768 | 384 × 384 | 192 × 192 | | 0.9927 | 2 |
| 1 | 0 | 768 × 768 | 384 × 384 | 192 × 192 | 96 × 96 | 0.9927 | 7 |
| 1 | 1 | 96 × 96 | | | | 0.9307 | 7 |
| 1 | 1 | 192 × 192 | | | | 0.9647 | 7 |
| 1 | 1 | 192 × 192 | 96 × 96 | | | 0.9700 | 7 |
| 1 | 1 | 384 × 384 | | | | 0.9700 | 7 |
| 1 | 1 | 384 × 384 | 96 × 96 | | | 0.9813 | 7 |
| 1 | 1 | 384 × 384 | 192 × 192 | | | 0.9750 | 7 |
| 1 | 1 | 384 × 384 | 192 × 192 | 96 × 96 | | 0.9813 | 7 |
| 1 | 1 | 768 × 768 | | | | 0.9697 | 7 |
| 1 | 1 | 768 × 768 | 96 × 96 | | | 0.9810 | 7 |
| 1 | 1 | 768 × 768 | 192 × 192 | | | 0.9787 | 7 |
| 1 | 1 | 768 × 768 | 192 × 192 | 96 × 96 | | 0.9810 | 7 |
| 1 | 1 | 768 × 768 | 384 × 384 | | | 0.9747 | 7 |
| 1 | 1 | 768 × 768 | 384 × 384 | 96 × 96 | | 0.9850 | 7 |
| 1 | 1 | 768 × 768 | 384 × 384 | 192 × 192 | | 0.9823 | 7 |
| 1 | 1 | 768 × 768 | 384 × 384 | 192 × 192 | 96 × 96 | 0.9850 | 7 |

**Table S5.** Validation accuracies of CAD with texture analysis in the public dataset.

| Normalization | Feature Selection | Image Resolutions | | | | Three-Category Accuracy | Optimal Machine Learning Algorithm |
|---|---|---|---|---|---|---|---|
| 0 | 0 | 96 × 96 | | | | 0.9867 | 7 |
| 0 | 0 | 192 × 192 | | | | 0.9873 | 7 |
| 0 | 0 | 192 × 192 | 96 × 96 | | | 0.9893 | 7 |
| 0 | 0 | 384 × 384 | | | | 0.9873 | 7 |
| 0 | 0 | 384 × 384 | 96 × 96 | | | 0.9897 | 7 |
| 0 | 0 | 384 × 384 | 192 × 192 | | | 0.9877 | 7 |
| 0 | 0 | 384 × 384 | 192 × 192 | 96 × 96 | | 0.9907 | 7 |
| 0 | 0 | 768 × 768 | | | | 0.9873 | 7 |
| 0 | 0 | 768 × 768 | 96 × 96 | | | 0.9913 | 7 |
| 0 | 0 | 768 × 768 | 192 × 192 | | | 0.9880 | 7 |
| 0 | 0 | 768 × 768 | 192 × 192 | 96 × 96 | | 0.9890 | 7 |
| 0 | 0 | 768 × 768 | 384 × 384 | | | 0.9897 | 7 |
| 0 | 0 | 768 × 768 | 384 × 384 | 96 × 96 | | 0.9890 | 7 |
| 0 | 0 | 768 × 768 | 384 × 384 | 192 × 192 | | 0.9877 | 7 |
| 0 | 0 | 768 × 768 | 384 × 384 | 192 × 192 | 96 × 96 | 0.9923 | 7 |
| 0 | 1 | 96 × 96 | | | | 0.9707 | 7 |
| 0 | 1 | 192 × 192 | | | | 0.9767 | 7 |
| 0 | 1 | 192 × 192 | 96 × 96 | | | 0.9787 | 7 |
| 0 | 1 | 384 × 384 | | | | 0.9653 | 7 |
| 0 | 1 | 384 × 384 | 96 × 96 | | | 0.9763 | 7 |
| 0 | 1 | 384 × 384 | 192 × 192 | | | 0.9757 | 7 |
| 0 | 1 | 384 × 384 | 192 × 192 | 96 × 96 | | 0.9803 | 7 |
| 0 | 1 | 768 × 768 | | | | 0.9650 | 7 |
| 0 | 1 | 768 × 768 | 96 × 96 | | | 0.9783 | 7 |
| 0 | 1 | 768 × 768 | 192 × 192 | | | 0.9773 | 7 |
| 0 | 1 | 768 × 768 | 192 × 192 | 96 × 96 | | 0.9803 | 7 |
| 0 | 1 | 768 × 768 | 384 × 384 | | | 0.9633 | 7 |
| 0 | 1 | 768 × 768 | 384 × 384 | 96 × 96 | | 0.9780 | 7 |
| 0 | 1 | 768 × 768 | 384 × 384 | 192 × 192 | | 0.9777 | 7 |
| 0 | 1 | 768 × 768 | 384 × 384 | 192 × 192 | 96 × 96 | 0.9827 | 7 |
| 1 | 0 | 96 × 96 | | | | 0.9867 | 7 |
| 1 | 0 | 192 × 192 | | | | 0.9873 | 7 |
| 1 | 0 | 192 × 192 | 96 × 96 | | | 0.9893 | 7 |
| 1 | 0 | 384 × 384 | | | | 0.9873 | 7 |
| 1 | 0 | 384 × 384 | 96 × 96 | | | 0.9897 | 7 |
| 1 | 0 | 384 × 384 | 192 × 192 | | | 0.9877 | 7 |
| 1 | 0 | 384 × 384 | 192 × 192 | 96 × 96 | | 0.9917 | 7 |
| 1 | 0 | 768 × 768 | | | | 0.9873 | 7 |
| 1 | 0 | 768 × 768 | 96 × 96 | | | 0.9907 | 7 |
| 1 | 0 | 768 × 768 | 192 × 192 | | | 0.9883 | 7 |
| 1 | 0 | 768 × 768 | 192 × 192 | 96 × 96 | | 0.9890 | 7 |
| 1 | 0 | 768 × 768 | 384 × 384 | | | 0.9893 | 7 |
| 1 | 0 | 768 × 768 | 384 × 384 | 96 × 96 | | 0.9893 | 7 |
| 1 | 0 | 768 × 768 | 384 × 384 | 192 × 192 | | 0.9880 | 7 |
| 1 | 0 | 768 × 768 | 384 × 384 | 192 × 192 | 96 × 96 | 0.9923 | 7 |
| 1 | 1 | 96 × 96 | | | | 0.9707 | 7 |
| 1 | 1 | 192 × 192 | | | | 0.9767 | 7 |
| 1 | 1 | 192 × 192 | 96 × 96 | | | 0.9777 | 7 |
| 1 | 1 | 384 × 384 | | | | 0.9653 | 7 |
| 1 | 1 | 384 × 384 | 96 × 96 | | | 0.9767 | 7 |
| 1 | 1 | 384 × 384 | 192 × 192 | | | 0.9757 | 7 |
| 1 | 1 | 384 × 384 | 192 × 192 | 96 × 96 | | 0.9803 | 7 |
| 1 | 1 | 768 × 768 | | | | 0.9643 | 7 |
| 1 | 1 | 768 × 768 | 96 × 96 | | | 0.9767 | 7 |
| 1 | 1 | 768 × 768 | 192 × 192 | | | 0.9773 | 7 |
| 1 | 1 | 768 × 768 | 192 × 192 | 96 × 96 | | 0.9810 | 7 |
| 1 | 1 | 768 × 768 | 384 × 384 | | | 0.9633 | 7 |
| 1 | 1 | 768 × 768 | 384 × 384 | 96 × 96 | | 0.9777 | 7 |
| 1 | 1 | 768 × 768 | 384 × 384 | 192 × 192 | | 0.9777 | 7 |
| 1 | 1 | 768 × 768 | 384 × 384 | 192 × 192 | 96 × 96 | 0.9820 | 7 |

© 2021 by the authors. Licensee MDPI, Basel, Switzerland. This article is an open access article distributed under the terms and conditions of the Creative Commons Attribution (CC BY) license (http://creativecommons.org/licenses/by/4.0/).
